# Selective Detection of Human Lung Adenocarcinoma Cells Based on the Aptamer-Conjugated Self-Assembled Monolayer of Gold Nanoparticles

**DOI:** 10.3390/mi10030195

**Published:** 2019-03-19

**Authors:** Ngoc-Viet Nguyen, Chun-Ping Jen

**Affiliations:** Department of Mechanical Engineering and Advanced Institute of Manufacturing with High-tech Innovations (AIM-HI), National Chung Cheng University, Chia Yi 62102, Taiwan; vietnn.mt@gmail.com

**Keywords:** lung cancer, circulating tumor cells, aptamer, gold nanoparticles (AuNPs), self-assembled monolayer (SAM)

## Abstract

This study established a microfluidic chip for the capture of A549 human lung circulating tumor cells via the aptamer-conjugated self-assembled monolayer (SAM) of gold nanoparticles (AuNPs) in the channel. AuNPs are among the most attractive nanomaterials for the signal enhancement of biosensors owing to their unique chemical, physical, and mechanical properties. The microchip was fabricated using soft photolithography and casting and molding techniques. A self-assembly method was designed to attach AuNPs, cell-specific aptamers, and target cells onto the desired area (i.e., SAM area). In this study, the gold microelectrode configuration was characterized by fluorescence microscopy and impedance measurements to confirm the important modification steps. Subsequently, several investigations with the proposed assay were conducted with different cell samples to determine the specific binding ability of the device for A549 adenocarcinoma cancer cells. This work has ensured a simple, convenient, selective, and sensitive approach for the development of biosensors for lung cancer detection during the early stages.

## 1. Introduction

Cancer is one of the most typical causes of human death around the world, with about 18 million new cases and up to 10 million cancer deaths each year. Lung cancer is the most frequent type in men and women, accounting for about 11.6% of total cancer diagnoses and 18.4% of total cancer deaths [1]. Thus, the early detection of cancer, particularly lung cancer, can help doctors choose appropriate and effective treatment methods. Cancer stages are associated with the molecular and morphological levels of rare malignant cells in the blood, such as circulating tumor cells (CTCs) and stem cells. These cells can generate differentiated cancer cell progenies that comprise a tumor [2]. For instance, the enumeration of CTCs detached from the primary tumor into the bloodstream can predict the stage of cancer metastasis progression [3]. However, existing chemotherapy and radiotherapy methods are not only expensive but also have difficulty detecting these rare cells [4,5]. Therefore, CTC isolation, detection, and quantification using sensitive and specific approaches can enhance the diagnostic accuracy of cancer.

Molecular probes and micro/nanofluidic techniques have been receiving great attention in the analytical and diagnostic fields. Typically, aptamer molecules are single-stranded deoxyribonucleic acids or RNA oligonucleotides that are isolated through a protocol known as the systematic evolution of ligands via exponential enrichment [6,7]. Since its first reports in the 1990s to date, aptamers have emerged as recognition biomolecules with high affinity and selectivity for various targets, such as proteins, amino acids, peptides, antibiotics, viruses, bacteria, whole or part of cells, and CTCs [8,9]. Aptamers have achieved several distinctive advantages due to their unique three-dimensional structure. They are stable in biological environments, have high structural flexibility, are nonimmunogenic, nontoxic, and smaller in size compared with an antibody. From these advantages, aptamers have been applied to many fields, such as therapeutics, diagnostics, microfluidics, biosensors, and bioanalytical fields [10].

Currently, electrical impedance-based biosensors have been preferably developed for the detection of living cells in microfluidic devices due to their simple fabrication, miniaturization capability, rapid response, and high sensitivity [11,12,13]. Among these biosensors, electrical impedance spectroscopy (EIS) measurement is a low-cost method that allows for the identification of the presence of biological cells. The changes of medium or materials inside the electric field region can be determined by measuring interfacial capacitance and resistance [14,15]. Impedance-based detection can not only quantify cell concentration in the medium but can also evaluate the electrochemical properties of the target cell in contact with the electrodes [16,17,18]. Aptasensors that combine impedance-based measurements and aptamers have a great potential for the classification of specific cells in many studies [19,20,21,22,23,24,25]. Several aptasensors have been reported for the detection of rare tumor cells, such as MCF-7 breast cancer [26], CT 26 colorectal carcinoma [20], and AGS human gastric cancer cells [27].

During the past few years, the field of biosensors has achieved remarkable advances due to the application of numerous nanomaterials, such as nanoparticles, nanotubes, nanowires, and nanostructures [28]. The optical, electrical, or mechanical features of nanomaterials are highly useful for signal generation and amplification. Through the combination with nanomaterials, the sensitivity of biosensors can be improved [29]. Gold nanoparticle (AuNP) is considered the most favored material type for the development of the aptamer-based sensors due to its electrochemical reactivity and conductivity properties. In addition, the conjugation of aptamers on AuNPs has also been well accomplished [28,30,31,32]. The electrochemical signal enhancement of the sensor using AuNPs can be classified into two approaches [33]. First, AuNPs are utilized as a label for signal amplification. Second, the sensing electrodes are enlarged through the attachment of AuNPs on the electrode surface. This second approach might increase the number of capture probes on electrodes, thus enhancing electrochemical signal intensity. In this study, a self-assembled monolayer (SAM) of AuNPs on the gold electrodes was designed and developed for cancer cell detection in the microfluidic channel [34]. These AuNP features have contributed to the design of simple, rapid, sensitive, and efficient aptasensors.

In this work, a simple microfluidic device was built for the capture of A549 human lung adenocarcinoma cells using a specific aptamer and the SAM of AuNPs, because A549 is known as the CTC cell line of the early stage in non-small cell lung cancer [22,35]. The improvement of early A549 cell detection was essential for increasing diagnostic accuracy. An assembly protocol was given to attach AuNPs at the investigation zone on the glass substrate. Target cell capture probes were prepared through conjugation of the thiol-labeled aptamers onto the AuNP layers in the channel. A sensing electrode structure at the SAM region was utilized for EIS measurements to demonstrate the binding events. The binding performance for target cells was expressed by monitoring the change of the fluorescence microscopic images. The obtained results may confirm a simple and rapid assay for the identification of cancer cell lines.

## 2. Materials and Methods 

### 2.1. Microfluidic Chip Design

**Figure 1:**
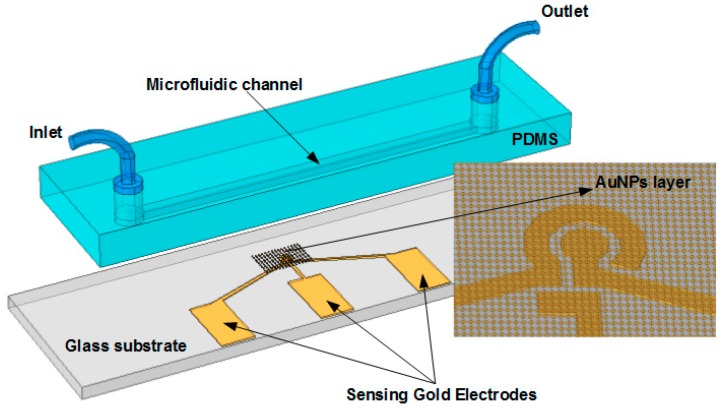
Schematic of the microfluidic device, including a glass substrate, a gold microelectrodes pattern and a polydimethylsiloxane (PDMS) channel. A self-assembled monolayer (SAM) of gold nanoparticles (AuNPs) was deposited to the location of the electrodes. The electrodes were used to identify the region of SAM, and the main binding events of the immobilization process. Aptamers were then conjugated onto the AuNPs layer to capture target cells in the microfluidic channel.

Figure 1 sketches the design of the proposed microchip, including a glass substrate, a gold electrode structure, and a straight microfluidic channel layer. The device was fabricated via soft photolithography and casting and molding techniques, as detailed in our previous publications [36,37]. The simple and sensitive microelectrode configuration patterned on the glass substrate was utilized to confirm the immobilization steps in the channel. A large area of SAM of AuNPs formed around the working electrodes was functionalized by the specific aptamers for trapping target cells. The target cells could be captured more easily around the sensing electrodes due to the larger SAM region. The channel made of polydimethylsiloxane (PDMS) was placed on the sensing location of the substrate, with length, width, and height of approximately 20 mm, 1 mm, and 50 µm, respectively.

### 2.2. Modification Procedure on the Substrate

**Figure 2:**
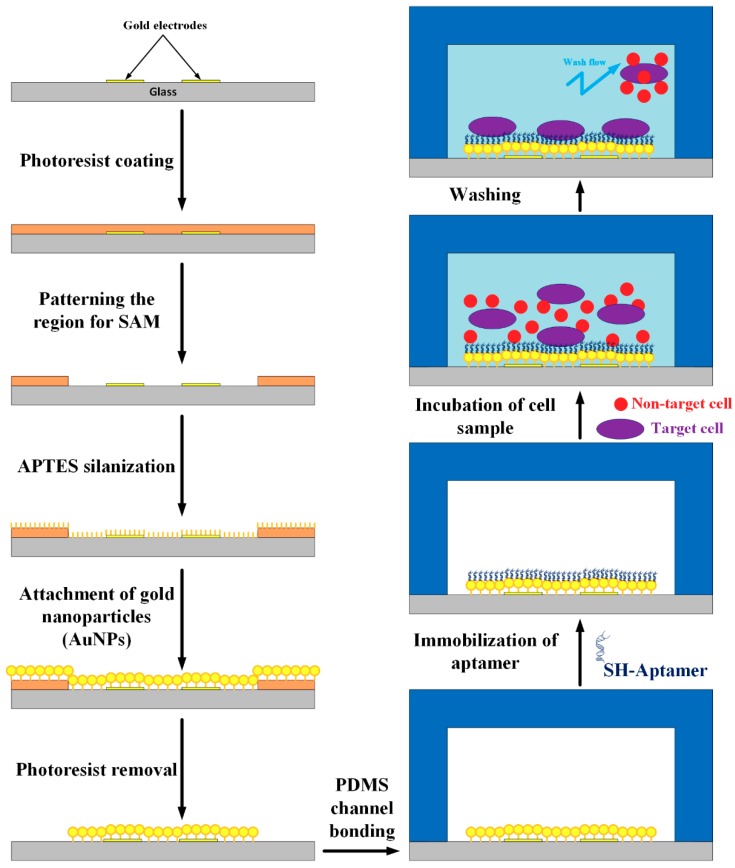
Illustration of aptamers modification procedure onto the SAM of AuNPs in the channel for binding of target cells.

The layer-by-layer assembly procedure onto the glass–gold substrate is illustrated in Figure 2. First, the square region for the SAM of AuNPs was created at the defined location of the electrodes by an S1818 photoresist layer via photolithography. The SAM area with dimensions of 1 mm × 1 mm was aligned completely in the channel. Salinization on the substrate surface was achieved using a (3-aminopropyl) triethoxysilane (APTES) mixture solution/H_2_O with a volume ratio of 1:1000 for 1 min. The APTES silanization and AuNPs self-assembly processes have been reported in our previous publications [34,38,39,40]. The obtained results indicated that the salinization duration of 1 min was suitable. The substrate was subsequently nitrogen-dried after being rinsed with deionized (DI) water and then reacted for 1 h in a 179 ppm AuNP solution. Then, the substrate was cleaned to remove the photoresist layer using acetone, methanol, and DI water and was then dried with nitrogen. After AuNP assembly in the designed region, the PDMS replica was bonded to the substrate using oxygen–plasma treatment (P[O_2_] = 500 mTorr, 50 s) in an O_2_ plasma cleaner. A PDMS prepolymer mixture was made by mixing PDMS prepolymer and curing agent in a 10:1 weight ratio. The mixture was poured and cured on the mold master for 2 h at 75 °C to replicate the patterned structure. After peeling off the PDMS replica, the inlet and outlet fluidic ports of the channel were created using a puncher.

Subsequently, the aptamer immobilization steps on the AuNP layers in the channel were performed. The DNA aptamer with 5′-thiol (5′-C6SH) modification was provided in a dried form, with a sequence of 5′-ACGC TCGG ATGC CACT ACAG GGTT GCAT GCCG TGGG GAGG GGGG TGGG TTTT ATAG CGTA CTCA GCTC ATGG ACGT GCTG GTGA C-3’. This thiol-labeled aptamer has a selective binding for A549 lung circulating tumor cells [41]. The 100-mM stock solution of the aptamer was prepared using a TE buffer (pH 8.0, 10 mM Tris, and 1 mM EDTA) and stored at −20 °C. A washing buffer solution containing 4.5 g of glucose, 5 mL of 1 M MgCl_2_ and 1 L of 10 mM Dulbecco’s phosphate-buffered saline (DPBS 1× at pH 7.4) without calcium and magnesium was stored at 4 °C for up to 3 months. A binding buffer was prepared by adding 1 mg of tRNA and 10 mg of bovine serum albumin into 10 mL of washing buffer. This buffer was then stored at 4 °C for up to 1 month. All aqueous solutions could be diluted with DI water (18.2 MΩ·cm) from a Direct-Q system (Milli-Q, Millipore Corporation, Billerica, MA, USA).

The channel was first washed with the washing buffer solution. The initial 100 mM SH-aptamer solution was diluted to an experimental concentration using the binding buffer. The refreshed aptamer solution was heated at 95 °C for 5 min and was left on a bench for 10 min at room temperature. Next, the channel was fully filled with the aptamer solution and then incubated for over 1 h. In practical surveys, various related parameters can be analyzed to establish the optimal conditions of the aptamer immobilization assay, such as media pH and temperature, incubation time, and aptamer concentration. In general, the response of the aptamer probes is gradually enhanced by increasing the concentration. In this work, the aptamer probes were normally operated in a media pH ranging from 7.0 to 7.4 at room temperature. The aptamer concentration of approximately 10 µM was selected in subsequent experiments. Finally, the cell sample was injected into the channel with an incubation time ranging from 1 to 5 min. The channel was then continuously washed by the buffer solution to remove nonspecific adsorbed molecules and to retain the target cells at the desired area. A flow rate of 10 µL/min was supplied into the microfluidic channel in all the experiments.

### 2.3. Cell Lines and Cell Culture

In this study, human epithelial adenocarcinoma cells, including A549 (human nonsmall cell lung cancer cell line) and HeLa (human cervical cancer cell line) cell samples, were used for the demonstrations of the proposed device. A549 and HeLa cell lines were cultured in Dulbecco’s modified eagle medium and minimum essential medium, respectively. The medium solutions were supplemented with 3.7 g of NaHCO_3_ per liter of medium, 10% fetal bovine serum, and 1% penicillin/streptomycin in a humidified atmosphere containing 5% carbon dioxide at 37 °C. The culture medium was replaced every 1 to 2 days. Prior to the experiments, the cells were gathered and segregated from the medium dishes via standard trypsinization. The cells were rinsed with the PBS buffer washing solution. The cell sample was resuspended in the binding buffer solution to procure a homogeneous cell suspension. The cell viability and concentration could be assessed by the trypan blue dye exclusion protocol and a hemocytometer with two counting grids.

### 2.4. Instrumentation

The solutions were injected into the channel of the chip using a syringe pump system (Model KDS 101, KD Scientific Inc., Holliston, MA, USA). Cell distribution in the sensing region was observed under an inverted fluorescence microscope (CKX41, Olympus, Tokyo, Japan) with a mounted CCD camera (DP71, Olympus, Tokyo, Japan) connected to a computer running Olympus DP Controller image software. Electrical impedance spectroscopy measurements were performed using a precision impedance analyzer (MICROTEST 6630, New Taipei City, Taiwan). The device could provide the excitation signals in large ranges of potential and frequency. The electrodes were driven by the impedance analyzer via coaxial cables. The measured data were collected and transferred to a computer for analysis. 

## 3. Results and Discussion

### 3.1. EIS Measurements in the SAM Immobilization Process

**Figure 3:**
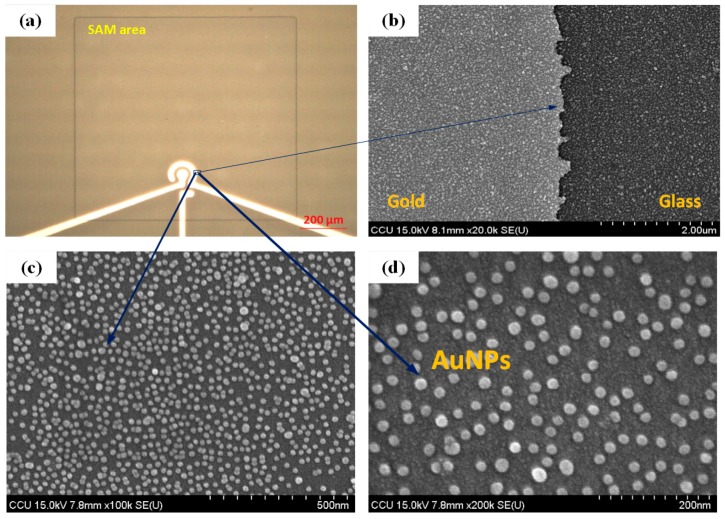
(**a**) A SAM area at the sensing electrodes after a fabrication process of spin coating photoresist, exposure, and development; (**b**–**d**) Scanning electron microscope (SEM) images of the self-assembled monolayer of AuNPs on the gold-glass substrate with the AuNPs concentration of 179 ppm at the magnifications of 20,000 times, 100,000 times, and 200,000 times, respectively.

In this work, the structure of gold microelectrodes was used to mark the desired SAM area and to reveal the stepwise modification process in the channel. It consisted of a center electrode with a diameter of 50 µm and an outer circular electrode of 30 µm in width. The gap between the two electrodes was 30 µm. Figure 3a shows the microscopic image of the SAM area around the electrodes. Gold beads with a mean diameter of 13 nm, purchased from Taiwan Advanced Nanotech Inc., were used in this study. The AuNPs diameter of 13 ± 1 nm was also chosen as the optimal size in our previous works for the protein preconcentrators [34,38,39,40]. Figure 3b–d shows the SAMs of AuNPs on a gold–glass substrate captured by a scanning electron microscopy system at magnifications of 20,000, 100,000, and 200,000 times, separately. These images confirmed that AuNPs could be attached to gold and glass surfaces with a high density. The subsequent experiments were conducted to demonstrate that A549 lung cancer cells can be trapped on the aptamer-coated AuNPs layer.

**Figure 4:**
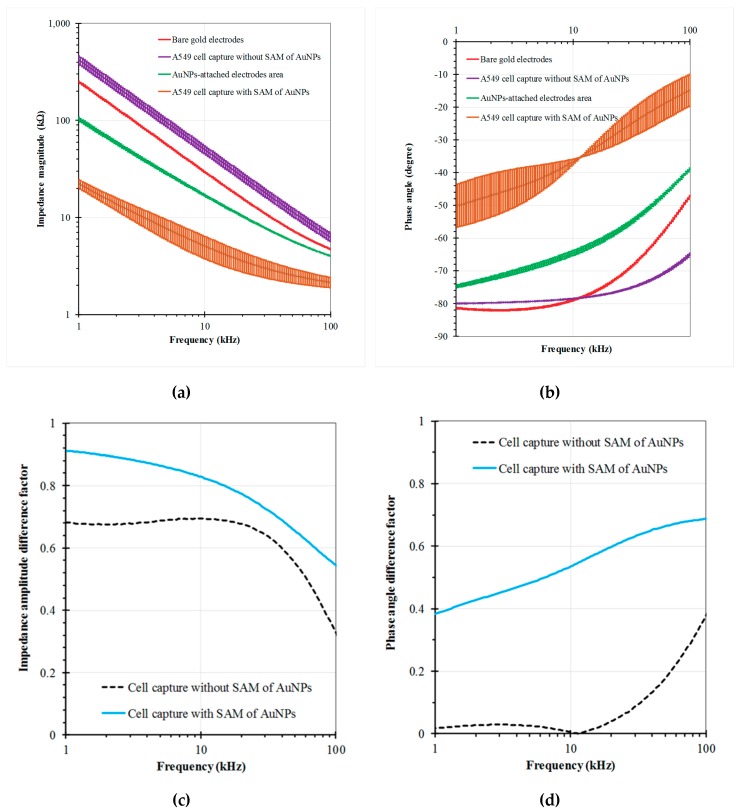
Impedance responses of the electrodes in the buffer microfluidic channel as a function of frequency in 4 measurements, respectively: the initial gold microelectrodes, the aptamer-modified gold electrodes without SAM of AuNPs after cell capture, the gold electrodes with SAM of AuNPs, and the modified electrodes with SAM of AuNPs after cell capture, with a voltage amplitude of 50 mV, and a frequency range from 1 to 100 kHz: (**a**) Impedance magnitude; (**b**) Phase angle; (**c**,**d**) Impedance and phase angle difference ratios of the cell-captured electrodes with respect to the initial electrodes in two cases, without and with SAM of AuNPs, respectively. The A549 cell samples at the same concentrations of 5 × 10^2^ cells/µL were used herein.

To evaluate the efficiency of the SAM assay, the EIS measurements were performed in the PBS buffer medium at an amplitude of 50 mV and with frequencies ranging from 1 kHz to 100 kHz. EIS responses were recorded at 125 points per decade. Fluidic flow in the channel was stopped during the measurements. Results that represent the average values of at least three separate experiments are shown in the form of amplitude Z (Figure 4a), phase angle θ (Figure 4b). A comparison of the EIS-based response signals from the electrodes in two cases was assessed in terms of the use and non-use of SAM of AuNPs. Figure 4 expresses the measured impedance values of the two electrodes in four investigations, including the initial gold electrodes without the SAM generation, the 10 µM aptamer-modified electrodes without SAM of AuNPs after cell capture and final washing, the gold electrodes with SAM of AuNPs, and the 10 µM aptamer-functionalized electrodes with the SAM of AuNPs after cell capture and final washing. The A549 cell samples at the same concentrations of 5 × 10^2^ cells/µL were used herein. The impedance magnitude decreased, whereas the phase angle increased with the increase in applied frequency. Besides, a distinct difference can be realized in the EIS graph trends in the two mentioned cases. For the case without SAM of AuNPs, the impedance magnitude of the aptamer-modified electrodes increased insignificantly compared with the original electrode at each frequency, whereas the phase angle was almost constant at low frequencies then slightly decreased at higher frequencies. In this case, aptamers only covered the surface of the gold microelectrodes without catching on the glass surface or the gap between the two electrodes. Several surveys have been presented in our previous report [37]. In the other case, the impedance amplitude decreased sharply during the modification process using AuNPs. The impedance value at each frequency point decreased by tens of kilo Ohms after the attachment of the AuNP layer and especially after aptamer incubation. The changes were observed clearly at low frequencies ranging from 1 kHz to 10 kHz. At such frequencies, the measured impedance values were high, whereas the phase angle values were low, indicating that the capacitive elements of impedance were dominant in a low frequency range. By contrast, the resistive components dominated at high frequencies. The immobilization process resulted in an increase in the conductivity of the electric field between the electrodes and a reduction in the measured impedance magnitude at each frequency point because of the envelopment of the aptamers and AuNPs on the gap and surface of the electrodes. The obtained results also indicated that the aptamers were successfully covered on the AuNP layer using the proposed SAM functionalization method. 

The performance of the SAM method was investigated on the basis of the EIS differential ratios in the investigated frequency range. The factors were defined in terms of amplitude difference factor $\Delta Z=\left|1-Z_{A}\left(f\right)/Z_{I}\left(f\right)\right|$ and phase angle difference factor $\Delta \theta =\left|1-\theta_{A}\left(f\right)/\theta_{I}\left(f\right)\right|,$ separately, where the indicators I and A are representative of the initial electrodes and the aptamer-modified electrodes at each individual frequency (f) after the cell capture process, respectively. Figure 3c,d shows the impedance variation in cases without and with SAM of AuNPs, respectively. In general, the coefficient lines with the use of the AuNPs were higher than those without the AuNPs in the impedance magnitude and phase angle analysis, especially in the low frequency range. Results indicated that the EIS signal with the use of AuNPs achieved a higher sensitivity with compared to without SAM of AuNPs. Furthermore, the aptamer-modified sensing electrodes could be stored in the buffer medium at 4 °C for up to 15 days. EIS responses still maintained more than 90% of their initial signal responses. Consequently, EIS was proven to be a potential tool to recognize the modification steps of the protocol.

In this study, we focused on the capture ability of the device for A549 lung cells using the aptamer-conjugated self-assembled monolayer of AuNPs. The electrochemical signal improvement for the impedance-based measurement of the sensing electrodes has been explored in the two cases of with SAM of AuNPs, and without SAM of AuNPs. The above experimental results showed that A549 target cells were trapped with high affinity and selectivity onto the aptamer-conjugated AuNP SAM in the microfluidic channel. However, the design still has some drawbacks in the application of the impedance measurement for cell detection. For instance, the CTC abundance in the real cell sample is extremely low. As a result, the extremely low numbers of the target cell could be captured into the electric field between the microelectrodes. Thus, the chip must be continuously improved in future works, including the optimization of the sensing electrode structure and the microfluidic channel design. In addition, the extension of the SAM layer area and application of the DEP-based cell manipulation will be proposed to manipulate the target cells onto the sensing electrodes conveniently [42,43,44,45]. Although its detection capacity was still limited, the microfluidic device exhibited many attractive features, such as biocompatibility, cost-effectiveness, simplicity, rapidity, high affinity, and selectivity toward the diagnosis of lung cancer cells.

### 3.2. Cell Specificity and Selectivity

**Figure 5:**
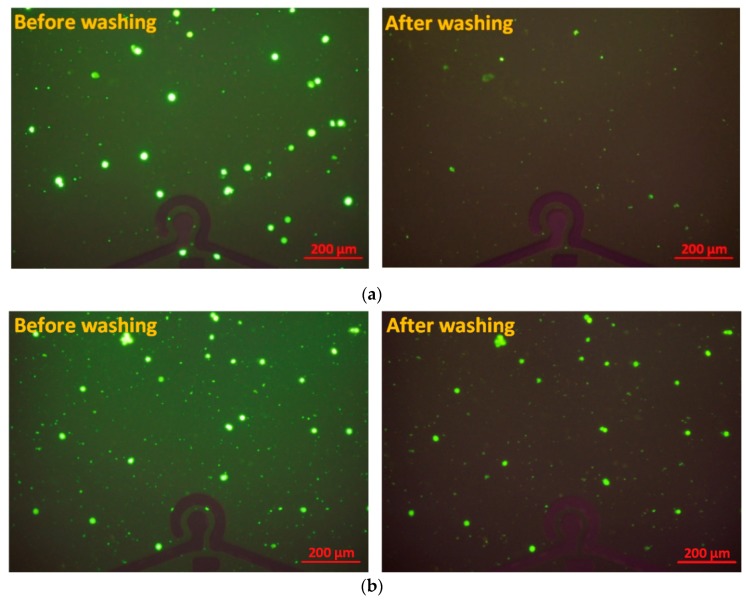
Fluorescence microscopic images of A549 cell samples during the washing and capture process onto the aptamer-modified electrodes area taken at the cell concentration of 5 × 10^2^ cells/µL: (**a**) Without SAM of AuNPs; (**b**) With SAM of AuNPs around the microelectrodes.

In this study, the A549 lung circulating tumor cell line was selected as the capture target of the aptamer. Prior to the experiments, A549 cells were stained using a standard fluorescence assay with Calcein green AM (Life Technologies, Carlsbad, CA, USA). Viable tumor cells were brightly fluorescent; thus, the number and viability of tumor cells could be verified. Following the immobilization of aptamers onto the sensing electrode region in the channel, A549 cell samples were pumped into the channel in the experimental repetitions. The cell sample solution at a defined cell concentration of 5 × 10^2^ cells/µL was fully filled in the channel. The cell sample was incubated in the channel before the final washing step using the buffer solution. The incubation time of the cell sample was explored in a range from 1 to 5 min with a step by step of 30 s. The target cell capture response of the aptasensor gradually increased with increasing incubation time. The target cell attachment still reached stability at 2 min. However, a longer incubation time could lead to a number of non-target cells also adhering onto the SAM layer. Thus, the incubation time of 2 min was chosen as the optimal incubation time of cell solution in these experiments. Figure 5 shows the fluorescence microscopic images of the A549 cell samples at the domain of the SAM area around the electrodes before and after washing by the syringe pump system for 3 min. The washing flow rate in the channel was set at 10 µL/min. The mean flow velocity in the microchannel was about 3.3 mm/s. The captured cells and aptamers might have been washed away at the higher speed range of the microfluidic flow. The images were obtained at the same objective scale and exposure mode established by the Olympus DP Controller image software. The fluorescence intensity was quantified using NIH ImageJ software. The efficiencies of cell capture were evaluated by calculating the fluorescence intensity of the SAM region at before and after the final washing step. Results indicated that the A549 cells were removed in the channel without the SAM of AuNPs on the electrode area, whereas they were still bound on the SAM of AuNPs area modified by aptamers with a cell binding coefficient over 90% at the cell concentration range from 10^5^ to 10^6^ cell/mL. These microscopic images also indicated the successful functionalization process of the SAM layers onto the gold–glass substrate on the microchip. 

**Figure 6:**
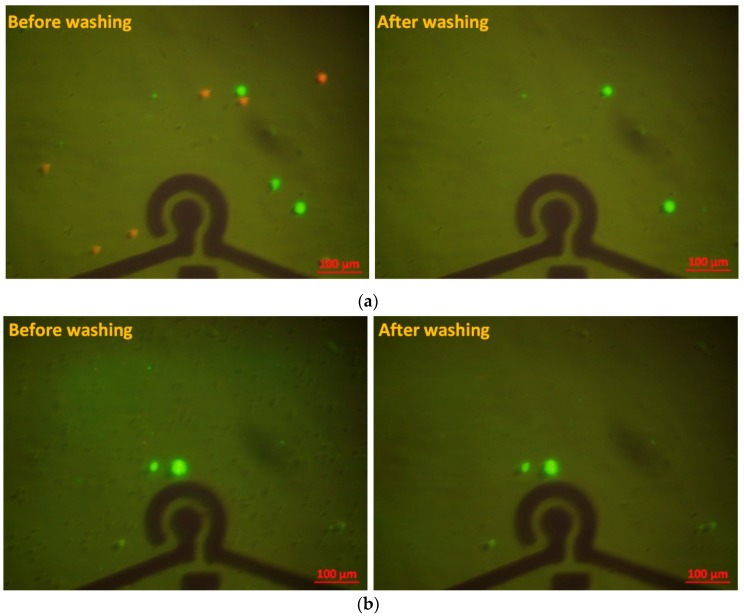
Fluorescence microscopic images of the mixture of cell samples around the aptamer-modified electrodes with the SAM of AuNPs area before and after the final washing step: (**a**) A549 cells (green) with the concentration of 5 × 10^2^ cells/µL, and HeLa cells (red) with the concentration of 10^3^ cells/µL; **(b)** A549 cells (green) at the concentration of 5 × 10^2^ cells/µL in 5% whole blood sample.

To estimate the affinity and specificity of the aptamer, several nontarget human cell lines were investigated previously, such as HeLa (human cervical cancer cells), MKN45 (human gastric cancer cells), and Caco-2 (human colorectal cancer cells) [37]. The aptamers with the same sequence were immobilized onto the surface of the gold electrodes. Results showed that nearly all the control cells were removed from the aptamer-modified electrodes, indicating the remarkably high specificity of this aptamer type for A549 line isolation among these human adenocarcinoma cells. To demonstrate the potential application of the device for the detection of A549 cells in complicated cell samples, A549 cells were mixed with HeLa cells and 5% whole blood sample (collected from healthy donors) in the buffer solution. Before the mixture of cells, A549 and HeLa cells were labeled by Calcein green and red-orange, respectively. To obtain the cell mixture, 500 µL of A549 cell solution at a concentration of 10^6^ cells/mL was combined with 500 µL HeLa cell solution at a concentration of 2 × 10^6^ cells/mL. Thus, the concentrations of A549 cells and HeLa cells were 5 × 10^5^ cells/mL and 10^6^ cells/mL, respectively. Similarly, 5 µL of whole blood was diluted in 995 µL of A549 cell solution at a concentration of 5 × 10^5^ cells/mL. The cell mixture samples were then assayed, as in the above process. As shown in Figure 6, most of the A549 cells were trapped onto the SAM section, whereas the other nontarget cells were washed away. These results confirm that the established device is a promising analytical tool with high selectivity for the capture and sensitive detection of A549 lung CTC cells.

## 4. Conclusions

The combination of AuNPs and aptamers can realize the excellent improvement in the sensitivity of the aptasensors. In this study, a simple microfluidic platform using cell-specific aptamers modified onto an AuNP layer was presented for trapping target cells. An identified zone of AuNPs was deposited on top of the glass substrate previously patterned with gold microelectrodes. Following the formation of the PDMS microfluidic channel on the sensing area, the thiol-terminated aptamers were injected and self-assembled to the AuNP surface through S–Au bonds. The measured impedance data indicated that the successful binding of the molecules was achieved by the proposed modification procedure, and the aptamers were effectively immobilized onto the SAM of AuNPs. In addition, the obtained fluorescence imaging results showed a highly selective capture performance of the experimental assay for A549 lung CTC cells. The fabricated microchip was also applied for the isolation of A549 cells in the whole blood samples. This study has enabled the optimization of channel and electrode designs as well as the development of aptasensors with excellent specificity, sensitivity, and stability for quantification and detection of cancer cells.
